# Walking onset: a poor predictor for motor and cognitive skills in healthy preschool children

**DOI:** 10.1186/s12887-021-02828-4

**Published:** 2021-08-27

**Authors:** Nadine Messerli-Bürgy, Tanja H. Kakebeeke, Andrea H. Meyer, Amar Arhab, Annina E. Zysset, Kerstin Stülb, Claudia S. Leeger-Aschmann, Einat A. Schmutz, Susi Kriemler, Jardena J. Puder, Simone Munsch, Oskar G. Jenni

**Affiliations:** 1 Department of Psychology, University of Fribourg, Fribourg, Switzerland; 2 Institute of Psychology, University of Lausanne, Lausanne, Switzerland; 3 Child Development Center, University Children’s Hospital Zurich, Zurich, Switzerland; 4 Children’s Research Center, University Children’s Hospital Zurich, Zurich, Switzerland; 5 Department of Clinical Psychology and Psychotherapy, University of Fribourg, Fribourg, Switzerland; 6 Department for Psychology, University of Basel, Basel, Switzerland; 7 Obstetric Service, Department Woman-Mother-Child, Lausanne University Hospital, Lausanne, Switzerland; 8 Epidemiology, Biostatistics and Prevention Institute, University of Zurich, Zurich, Switzerland

**Keywords:** SPLASHY, Onset of walking, Development, Preschool, Cognition

## Abstract

**Background:**

The onset of walking is thought to be an indicator of early development. However, evidence is mixed and clear data on this relationship at preschool age is missing. The study aimed at investigating if walking onset and motor and cognitive development in preschool children are related.

**Methods:**

A total of 555 children (mean age 3.86 years) of the Swiss Preschoolers’ Health Study SPLASHY were tested twice at their childcare center (at baseline and one year later). Motor skills and cognitive skills were assessed by standardized testing procedures and parents were asked to provide information on walking onset of their child.

**Results:**

Late onset of walking was related to poorer motor skills (fine motor skills, static and dynamic balance (all *p* < 0.003)) and poorer cognitive skills (selective attention and visual perception (*p* = 0.02; *p* = 0.001) in late preschool age.

**Conclusions:**

For children with late walking onset a close monitoring of their development in the regular pediatric child health visits may be reasonable.

Trial registration: ISRCTN41045021.

## Background

The onset of independent walking in childhood is a measure that parents tend to relate to the developmental speed of their child. Advanced children may then be accredited to a faster development in comparison to children who start to walk late. This assumption is not quite correct. Early motor skills have been related to better general development in children with a medical record (brain injury etc.) or prematurity [1], but only weakly to the development of healthy children [2–5].

Since about 50 years the study of the onset of independent walking has been placed in a wider ecological perspective [6–8]. Motor skill development was explained by means of the interplay between constraints from the task, the organism and the environment that specifies the optimal pattern of motor coordination and motor control [8]. This means for example that long legs might lead to earlier walking due to its advantage for the walking activity [9]. Further, the amount of practice leads to a gain of stronger muscles in the legs, thereby improving balance [10] and maintaining a coordinated leg movement [11] which results in better and earlier independent walking. It was shown by Clearfield, Osvorne & Mullen [12] and Clearfield [13] that the onset of walking was accompanied by a transition of passive observer to active walking participants. This implies that the child begins to actively investigate and experience the world around him with the onset of walking [13] as also described in the concept of ‘enabling’ according to Adolph & Hoch [14].

This indicates that the onset of walking is an important step in the motor domain being related to the social and perceptual domains of a child [12]. In this study the impact of the onset of walking is investigated not only on the motor, but also the cognitive domain.

Previous studies showed consistent associations of early walking onset and better motor development later in healthy children, but effects were generally small [3, 5, 15]. In contrast, findings about the association of early walking onset and better motor and cognitive development at younger age are rather mixed. Whereas Ghassabian et al. [2] reported a relationship between motor milestones and cognitive development in children aged 4 years, Jenni et al. [3] did not find significant associations between motor skills and intelligence levels at the age of 7 years. These differences might be explained by the different age periods considered in the two studies. Slow developers with late walking onset might have “caught up” on cognitive development at school age as positive changes of cognitive skills (especially related to inhibitory behavior) are expected up to school age [15] and poor development at an early age can be compensated up to that age. The two studies investigated children at the specific ages of 4 and 7 years and evidence is lacking for an age range covering the whole preschool age period. This study aimed to assess the relationship between walking onset and both motor and cognitive skills in healthy preschool children of 2–6 years at two different time points 12 months apart.

## Methods

### Study sample and design

The study sample consisted of 555 children of the Swiss Preschooler’s Health Study (SPLASHY), a multi-site prospective cohort study recruited in 84 childcare centers of Switzerland (ISRCTN41045021; for details see Messerli-Bürgy et al. [16]). The study was approved by all local ethical committees (No 338/13 for the Ethical Committee of the Canton of Vaud as the main ethical committee (site of Lausanne). Further the ethical committees of all other study sites approved the study protocol including Zurich, Aargau and Bern. The study protocol is in accordance with the Declaration of Helsinki.

Details of the study design and the overall objectives have been previously described [16]. Parents were asked to let their children participate in the study and provided written informed consent. Children were excluded with an age above 6 years and below 2 years. Children were assessed at two time points (baseline and 12 months later) at the childcare centers and further data was collected through parental questionnaires. All assessments were carried out according to standardized procedure guidelines in parallel at all sites (Zurich, Fribourg/Bern and Lausanne). Adherence to procedure guidelines was guaranteed by the specific expertise of the principal and co-investigators via multiple site visits, videotapes and supervision of contributors.

### Assessment

#### Onset of walking

Parents were asked to record the age in months at which the child achieved his first independent steps.

#### Motor skills

The Zurich Neuromotor Assessment (ZNA) [17] was used to assess motor skills of all children. The ZNA is a standardized motor test procedure based on timed performance and quality of movements (intensity of associated movements, which are unintended movements occurring on the contralateral side at the same time). The test includes fine (i.e. with toys) and pure motor (i.e. without toys, repetitive, alternating and serial movements) tasks of the hands (during which the intensity of associated movements were measured), static (standing on one leg) and dynamic balance tasks (hopping on one leg, going on a straight line, jumping sideward and running) [17]. All data was video-taped and analyzed by experts in a second step. Tasks were grouped on five components (fine motor skills, pure motor skills, static balance, dynamic balance and associated movements). The first four components were assessed according to the standards of the ZNA. Further, associated movements were gathered for the hands when the contralateral side was active. This last component provides information about the quality of movements and represents inhibitory control of motor skills. In order to make comparisons between components, the components are expressed as standard deviation scores [17].

#### Cognitive skills

Four cognitive subtests (visual perception, selective attention, visuospatial working memory and figural reasoning) of the Intelligence and Development Scales-Preschool (IDS-P) [18] were used to assess cognitive skills of the children. Maximum score (raw data) differed in each subtest and were 40 points for visual perception (arranging cards printed with pencils of varying lengths in a sequence), 72 points for selective attention (cards with drawings of ducks with specific characteristics need to be sorted out), 10 points for visuo-spatial working memory (geometric figures presented for a short time period need to be recognized afterwards in a selection of more figures), and 12 points for figural reasoning (visually presented geometric figures need to be replicated with wooden rectangles and/or triangles). Raw data were then transformed into age-adapted scores according to the official manual of IDS-P [18]. Further, inhibitory control was measured by the Statue test of the Neuropsychological Assessment for Children (NEPSY) battery [19] (range of 0–30 points). Assessments were video-taped and coded by experts.

### Covariates

*Socioeconomic status (SES)* was calculated by using the International Socio-Economic Index (ISEI) [20]. The highest ISEI value of maternal or paternal ISEI value was used for further calculation.

*Perinatal risks* were calculated by a sum value of preexisting perinatal stress conditions (range of 0–6 points). Parents were asked to complete the questions on child birth weight, pregnancy complications, birth complications and prematurity. All stress conditions were coded according to increased risk such as low child birth weight (< 2500 g) according to World Health Organization [21], existence of *pregnancy complications* (“Did you have any complications during pregnancy of your child (e.g. vaginal bleeding, suspected low fetal growth, premature labor pains, corticosteroid treatment before early birth (to increase maturation of the baby’s lungs), use of medication to reduce labor pains”), existence of *birth complications* (“Did you have any birth complications during birth or early after giving birth?”), existence of *prematurity* (“Did your child stay at the neonatal clinic?”), *maternal smoking during pregnancy* and *paternal smoking during pregnancy* (“Did you smoke during pregnancy?”).

#### Procedure

All testings were performed at the childcare centers by the research teams, trained and supervised by the professors being experts in their fields. In the second year, the training sessions were rehearsed in order to keep testing procedures standardized and supervised by the experts.

### Statistics

Multilevel models calculated by R software package [22, 23] were used to analyze the data. Hierarchical levels were the two waves nested within children, nested within child care centers. We used a random intercept. Fixed effects were walking onset, wave and the interaction between the two, plus the two covariates SES and number of negative perinatal conditions. The interaction term thereby tested whether the association between walking onset and the respective outcome differed between the two waves. For each outcome a separate analysis was run.

## Results

At the first assessment time point children had a mean age of 3.86 years (m/f: 293/262; 53% vs. 47%) and were 12 months older at the second time point. The children had a mean SES level of 62.54 (SD 15.5). Mean time point of walking onset was 13.26 months (SD 2.36, range 8–26). A total of 156 children experienced any kind of perinatal risk whereof 105 experienced one perinatal risk, 28 two, 14 three and 9 experienced four perinatal risks. Levels of motor skills and cognitive skills are presented in Table 1.

**Table 1:** Descriptives of motor and cognitive skills

	Wave 2014	Wave 2015
	M (SD)	M (SD)
*Motor skills*		
Fine motor	0.08 (1.01)	0.30 (1.00)
Pure motor	0.13 (1.16)	0.56 (1.10)
Static balance	0.09 (0.97)	0.23 (1.01)
Dynamic balance	0.01 (1.03)	0.11 (1.07)
Associated movements	−0.13 (1.01)	− 0.12 (0.95)
*Cognitive skills*		
Visual perception	10.10 (2.67)	23.60 (6.76)
Selective attention	9.27 (2.73)	38.54 (12.56)
Visuo-spatial memory	9.27 (3.21)	5.22 (2.36)
Figural reasoning	7.49 (2.60)	4.45 (3.14)
Inhibitory control	2.68 (1.70)	3.21 (1.73)

Note: All motor skills components are expressed as standard deviation scores and cognitive skills components as age-adjusted mean scores

Analyses revealed a negative relationship between walking onset and fine motor skills, being significant in the first but not in the second wave of assessment (Table 2). Thus, children with earlier walking onset showed higher levels of fine motor skills during early preschool age, and kept doing so during the preschool age period, as the difference between the regression coefficients among the two waves were not significant. Early walking onset was also negatively related to static and dynamic balance in both years of assessment within the preschool period (Table 2). Children with early walking onset showed higher levels of static and dynamic balance at early preschool age and were still doing better one year later. The differences in the regression coefficients between the first and second wave for static and dynamic balance were not significant, which implies that the impact of early walking on these two parameters did not change over the two waves. The relationship between early walking onset and pure motor skills significantly differed between the two waves, being negative in the first and positive in the second year. Thus, the earlier children walked, the higher their performance on pure motor skills in the first and the lower in the second year. Further, early walking onset was not related to associated movements at either wave.

**Table 2:** Regression coefficients for the relationship of walking onset and several outcomes (motor skills, cognitive skills) at each of two waves and between them

	First Wave			Second Wave			Difference between second and first wave		
	Coeff	SE	P	Coeff	SE	P	Coeff	SE	P
*Motor skills*									
Fine motor	−0.861	0.279	**0.002**	−0.575	0.297	0.053	0.286	0.295	0.33
Pure motor	−0.524	0.318	0.11	0.623	0.337	0.07	1.147	0.419	**0.006**
Static balance	−0.873	0.287	**0.003**	−1.162	0.299	**< 0.001**	−0.289	0.314	0.36
Dynamic balance	−1.316	0.301	**< 0.001**	−1.361	0.313	**< 0.001**	−0.045	0.341	0.896
Associated movements	0.131	0.313	0.68	0.015	0.361	0.97	−0.116	0.411	0.78
*Cognitive skills*									
Visual perception	−0.292	1.501	0.85	−3.556	1.549	**0.02**	−3.264	2.043	0.11
Selective attention	−1.613	2.594	0.53	−8.857	2.646	**0.001**	−7.244	3.432	**0.04**
Visuo-spatial memory	−0.703	0.842	0.41	−0.309	0.857	0.71	0.394	1.088	0.71
Figural reasoning	−0.605	0.837	0.47	−1.354	0.849	0.11	−0.749	1.005	0.56
Inhibitory control	0.079	0.505	0.88	−0.511	0.527	0.33	−0.591	0.602	0.33

Note: model 1: analyses controlled for SES; results did not differ when analyses were not controlled for perinatal factors

**Fig. 1:**
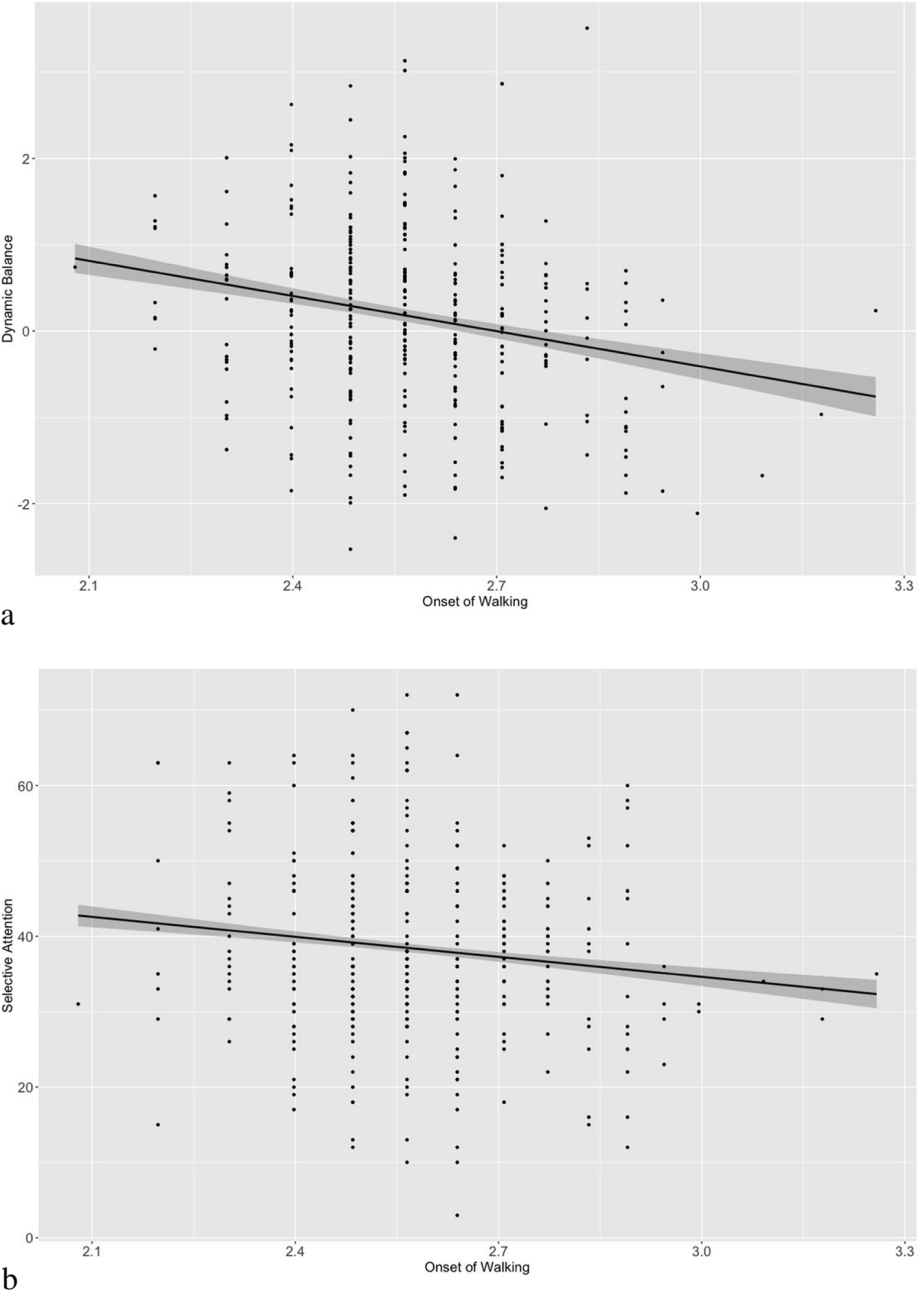
Scatter plots of the consistent relation between onset of walking and cognitive skills at late preschool age (**a.** the relation of onset of walking and dynamic balance; **b.** the relation of onset walking and selective attention)

Regarding cognitive skills, there was a negative relationship between time of walking onset and both visual perception and selective attention, being significant only in the second, but not in the first year. The difference in the regression coefficients was thereby significant for selective attention, but not for visual perception. In other words, children with early onset of walking showed higher levels of both visual perception and selective attention, especially at the second wave, and the strength of this relationship between the two waves was particularly pronounced for selective attention. Finally, early walking onset was not related to visuo-spatial memory, figural reasoning, or inhibitory control at both waves. Figure 1 shows the associations between early walking onset and dynamic balance (Fig. 1a) and selective attention (Fig. 1b).

## Discussion

The onset of walking has often been discussed as a potential predictor of optimal motor and cognitive development [3, 5, 15, 24, 25]. Using two separate waves that were one year apart, we investigated the impact of walking onset on motor and cognitive skills in preschool children aged 2–6 years. We found that the walking onset predicted specific aspects of motor skills at preschool age. Additionally, the walking onset was significantly associated with two aspects of cognitive skills (visual perception and selective attention). However, this association was limited to the second wave. Children who walked earlier scored better on visual perception and selective attention. There was no association between onset of walking time and inhibitory control, neither at the first nor at the second wave.

Onset of walking was related to fine motor skills, static balance and dynamic balance at the age of 2–6 years. This is in line with the results of Jenni et al. [3] who studied different milestones such as ‘sitting without support’ and ‘independent walking’. They investigated the relation of these motor developmental milestones with overall motor performance at the age of 7 to 18 years. They found an association with dynamic balance tasks in these school-aged participants [3]. In this study, the tasks related to dynamic balance scores consisted of hopping on one leg, going on a straight line, jumping sidewards and running similar to those of Jenni and colleagues [3]. In 2 of these 4 tasks, the child has to work against gravity and fulfilling these tasks reflects the child’s ability to produce force. As these tasks demand similar force of the upper leg to compete gravity as a walking task at an infant’s age, the associations of dynamic balance over the investigated age period of 3 years, 4 years (this study) and 7 years [3] are not surprising. There is evidence that postural control and enough leg power are needed as prerequisites for normal motor development [8, 11, 14], but extensive practising supports a further improvement of muscle strength. Therefore, children with early walking onset might have had more opportunities to practice and to develop a certain level of strength in their legs.

Focusing on different aspects of motor skills, we further found associations of walking onset and fine motor skills as well as static balance over both years of assessment. To our knowledge, these relationships have not been investigated in any other study before and therefore comparison with similar results is limited. However, we understand that fine motor skills and static balance may both contribute to the general motor skills at preschool age in this study, but their levels at school age are obviously less associated to the onset of walking [3].

In our study, early walking onset predicted high visual perception skills and high levels of selective attention at a later preschool age, but not at early preschool age. These distinct results of early and late preschool age were not expected, but findings on the predictive value of motor milestones have also shown mixed results in the literature before. Some studies found similar results, if slow developers were included [15] and if analyses were not controlled for negative perinatal conditions [2]. However, when considering a healthy sample, early motor development in infancy had no such strong impact on cognitive skills at preschool age (4 years) or at school age (7–16 years) in cohort studies [2, 3]. A potential association of independent walking with executive functioning was only reported in a rather small sample of adults [15], but those analyses were not controlled for perinatal conditions or particularly slow developing children. In our study, controlling for perinatal risks did not change anything to our results, although it needs to be considered that the threshold for mainly birth complications or perinatal risks was kept low considering caesarean sections (emergency or elective), use of forceps and vacuums, preeclampsia conditions, high blood loss and breathing problems of the child as perinatal risk conditions which resulted in a higher prevalence of perinatal problems than usually reported in other studies, but might not have caused similar developmental problems as would be expected in children with severe and life-threatening birth conditions. We have to add here, that there was no sign of clearly slow developers taking part in our study as others had included, but potentially certain differences in cognitive skills might only be visible at a point when full development is expected or can be understood as overdue. We hypothesize that this might be the case for visual perception which is known to achieve the level of an adult already at the age of 2–4 years [25] and complete binocular vision is attained during later preschool age (4–6 years) [25]. This developmental pattern may partly explain the results of these investigations as the relationship is expected to occur at a later age. Similarly, there is evidence that attention control becomes more flexible during the preschool age and therefore selective attention is still developing [26] and due to this late maturation strong differential changes can only be expected at the later period of preschool age.

As many others, we think that development is primarily a dynamic process [8, 14]. An organismic constraint for the first steps (or a physical prerequisite) is a strong lower leg muscle power and mature neuromuscular activation. Strong leg muscle power is needed for walking onset and similarly for dynamic and static balance tasks at older age. Having strong muscles is advantageous for the onset of walking and also for the later static and dynamic balance tasks as was shown in this study. This relationship between onset of walking and static and dynamic balance is still there at a later age as was shown by Jenni et al. [3] but less strong.

The high association between onset of walking, selective attention and visual perception could be explained by the fact that some maturational processes are obviously blooming at a later and not at an early preschool age. These maturational processes for inhibitory tasks (associated movements and performance on the statue task) did not take place within the study period, as we did not find any significant associations with the onset of walking. However, cognitive skills were not assessed at toddler’s age and data is only relying on the motor domain for that early time period. Assessment of visual perception and selective attention at the onset of walking is missing and therefore the association of motor and cognitive skills not proven. A further limitation of this study is that during the time period that we were testing, we were not able to capture the dynamic processes of inhibitory tasks (associated movements and performance on the statue task) and we assume that this might have influenced the lack of association between inhibitory tasks and the onset of walking.

## Conclusions

In this study, onset of walking predicted specific aspects of motor skills and of cognitive skills at preschool age. As the developmental stage of motor and cognitive skills are related to a generally healthy development of children at preschool age, we conclude that for children with late walking onset a close monitoring of their development in the regular pediatric child health visits may be warranted.

## Data Availability

The datasets generated and/or analyzed during the current study are not publicly available due the fact that participants were not asked at that time to provide consent on open data, but are available from the corresponding author on reasonable request.

## Abbreviations

SES: socioeconomic status
SPLASHY: Swiss Preschoolers’ Health Study
